# Artificial intelligence use as a key predictor of clinical performance in nursing students: a cross-sectional study from Iran

**DOI:** 10.1186/s12909-025-08454-3

**Published:** 2025-12-13

**Authors:** Rasool Paygozar, Noorollah Tahery, Samaneh Dehghan Abnavy

**Affiliations:** 1 Nursing Midwifery Research Center, Nursing and Midwifery School, Abadan University of Medical Sciences, Abadan, Iran; 2 Abadan University of Medical Sciences, Abadan, Iran; 3 Department of Operating Room, Community-Oriented Nursing Midwifery Research Center, Nursing and Midwifery School, Shahrekord University of Medical Sciences, Shahrekord, Iran

**Keywords:** Artificial intelligence, Nursing education, Clinical competence, Educational technology, Iran

## Abstract

**Background:**

The integration of artificial intelligence (AI) into healthcare education is rapidly evolving, yet its impact on clinical performance among nursing students remains underexplored, particularly in resource-constrained settings.

**Objectives:**

This study aimed to investigate the relationship between AI use and clinical performance among undergraduate nursing students, while controlling for key demographic variables.

**Methods:**

A cross-sectional study was conducted with 134 undergraduate nursing students from Abadan University of Medical Sciences, Iran, in 2024. Data were collected on AI use (Artificial Intelligence in Nursing Questionnaire), and Clinical Performance Questionnaire (CPQ). Data were analyzed using IBM SPSS Statistics (v26). Descriptive statistics, Pearson correlation, multiple linear regression, and univariate general linear modeling (GLM) were employed.

**Results:**

AI use demonstrated a significant positive correlation with overall clinical performance (*r* = 0.424, *p* < 0.001). In the multiple regression model, AI use was the only significant predictor of clinical performance (β = 0.425, *p* < 0.001), explaining 21.1% of the variance (*R²* = 0.211). Demographic variables (gender, academic term, age level) were non-significant. A univariate GLM confirmed a significant main effect for AI use (*F*(1,111) = 19.672, *p* < 0.001), independent of all demographic factors. Simple linear regressions revealed that AI use significantly predicted performance across all clinical subscales, with the strongest effects in Research (*R²* = 0.166), Patient-Centered Care (*R²* = 0.146), and Personal Management (*R²* = 0.127).

**Conclusion:**

AI use is a robust and independent predictor of clinical performance among nursing students. These findings underscore the transformative potential of AI in clinical education and advocate for the systematic integration of AI literacy into nursing curricula to enhance evidence-based practice, critical thinking, and patient-centered care.

## Introduction

In the current era, artificial intelligence (AI) is rapidly evolving in various scientific and medical fields. In nursing education, AI has the potential to accelerate the learning process and improve students’ clinical skills, allowing them to practice in simulated environments with zero risk. This technology can significantly improve the quality of clinical education, clinical decision-making, and practical capabilities of nursing students [1].

In many educational systems, there is limited opportunity for nursing students to gain practical experience in clinical settings. Artificial intelligence can compensate for this deficiency by providing advanced simulations and preparing students for real-world situations. AI-based simulations can also help nursing students make quick and accurate decisions in critical situations, while avoiding dangerous mistakes. Integrating artificial intelligence into nursing education programs can help improve the quality of education as a complementary tool. The use of advanced simulations, interactive learning environments, and decision support systems can improve the learning process [2, 3]. The healthcare education landscape has undergone profound transformation through artificial intelligence (AI) integration, particularly in nursing education. AI technologies have demonstrated remarkable potential to enhance clinical training through various innovative applications. Virtual patient simulators now enable nursing students to practice complex clinical scenarios with unprecedented realism, while intelligent tutoring systems provide personalized feedback tailored to individual learning needs. These advancements have been shown to significantly improve clinical skill acquisition, with recent studies reporting 40% greater skill retention compared to traditional methods [4, 5].

The benefits of AI in nursing education extend beyond skill development. AI-powered analytics systems can track student performance with remarkable precision, identifying knowledge gaps that might otherwise go unnoticed. Such systems have been associated with a 35% reduction in medication errors during clinical training [6, 7]. Furthermore, adaptive learning algorithms create customized educational pathways that accommodate diverse learning styles, potentially revolutionizing how nursing competencies are developed and assessed [7].

Despite these promising developments, significant challenges persist, particularly in resource-limited settings. Many nursing schools in developing nations face substantial barriers to AI adoption. Infrastructure limitations remain a critical concern, with recent surveys indicating that 58% of Middle Eastern nursing programs lack the necessary technological foundation for effective AI implementation [8–11]. Additionally, cultural factors and varying levels of technological literacy among both students and faculty present unique challenges that must be addressed [10, 11].

The disparity in AI adoption between developed and developing nations is striking. While approximately 83% of European nursing schools have integrated AI tools into their curricula, comparable systems are present in only about 17% of Middle Eastern institutions. This gap highlights the urgent need for context-specific research to guide AI implementation in different educational settings [12, 13].

To date, research on AI in nursing education within the Middle East remains limited and largely qualitative or conceptual. While studies have explored AI readiness [10], acceptance [11], and curricular challenges [9, 14], no empirical study from Iran or neighboring countries has quantitatively investigated the direct association between AI engagement and clinical performance among nursing students. This knowledge gap underscores the novelty and contextual relevance of the present study.

In Iran, where this study was conducted, AI adoption in nursing education remains in its early stages, with current estimates suggesting penetration in fewer than 15% of nursing programs [9, 14]. This study seeks to address this knowledge gap by examining the relationship between AI integration and clinical proficiency among Iranian nursing students This study is guided by the Technology Acceptance Model (TAM) [15], which posits that technology adoption is primarily driven by perceived usefulness (PU) and perceived ease of use (PEOU). In the context of nursing education, we conceptualize AI use as a behavioral outcome influenced by these core TAM constructs. Specifically, the AI Use scale (AINQ) assesses dimensions that map onto TAM: AI inclination (AIP) and AI-based decision-making (AIDM) reflect perceived usefulness, while AI skills (AIS) and AI infrastructure (AIB) relate to perceived ease of use.

Based on TAM and prior evidence, we hypothesize that higher self-reported AI use is positively associated with clinical performance, independent of demographic factors. Accordingly, the research questions are:

RQ1. Is there a significant positive correlation between AI use and overall clinical performance among undergraduate nursing students?

RQ2. Does AI use significantly predict clinical performance after controlling for gender, academic term, and age?

RQ3. Which clinical performance domains (e.g., research, patient-centered care) are most strongly associated with AI use?

The significance of this study lies in its potential to inform the development of culturally appropriate, resource-sensitive strategies for AI implementation in nursing education. By examining these relationships in the Iranian context, we aim to contribute valuable insights that could enhance clinical training while respecting local educational traditions and resource constraints. Our findings may help bridge the current divide in AI adoption between developed and developing nations, ultimately improving nursing education outcomes across diverse settings.

## Methods

### Study design and setting

This cross-sectional analytical study was conducted at the School of Nursing, Abadan University of Medical Sciences, Iran, from December 2024 to March 2025 (corresponding to *Dey–Esfand* 1403 in the Persian calendar). The setting included both classroom and simulation laboratory environments on the university campus.

### Participants and sampling

The target population comprised all undergraduate nursing students enrolled in semesters 3 to 6 (*N* = 141). A census sampling approach was adopted, inviting all eligible students. Inclusion criteria were: (1) active enrollment in the nursing program, and (2) voluntary informed consent. Students in semesters 7 and 8 were excluded because they were engaged in full-time external clinical internships throughout the data collection window, limiting their availability and physical access to university-based data collection channels. Their inclusion could have introduced non-response bias or compromised data quality due to inconsistent survey administration conditions.

### Data collection procedures

Data were collected through a mixed-mode approach to maximize response rate and accessibility:

- In-person mode: Paper-based questionnaires were administered during scheduled classroom sessions (for semesters 3–4) and simulation skill labs (for semesters 5–6). Instructors temporarily left the room during completion to ensure participant anonymity and reduce social desirability bias.
- Online mode: For students absent from on-campus sessions or undertaking remote rotations, a secure, password-protected online survey (hosted on Google Forms) was distributed via (1) the official university email list and (2) verified WhatsApp class group administrators. Two reminder messages were sent over a 7-day period.

All procedures were supervised by the first author (RP), with two trained research assistants assisting in paper-based administration. Participation was entirely voluntary and anonymous.

### Instruments

Two validated, self-report questionnaires were used in their Persian-translated versions (see *Translation and Validation* below):

1. Clinical Performance Questionnaire (CPQ).
2. Developed by Ebadi et al., this 44-item instrument assesses clinical performance across five domains: care management, academic competence, personal management, patient-centered care, and research/knowledge development. Items use a 5-point Likert scale (*1 = never* to *5 = always*). Total scores range from 44 to 220 (higher = better performance), with internal consistency in this study: Cronbach’s α = 0.89 [16].
3. Artificial Intelligence in Nursing Questionnaire (AINQ).
4. Adapted from Chen et al. (2022), this 22-item scale measures AI use across five dimensions: AI management (AIM), AI-based decision-making (AIDM), AI infrastructure (AIB), AI skills (AIS), and AI inclination (AIP). Responses use a 5-point Likert scale. Subscale scores are mean values (1–5); AITOTAL is the sum of all items (range: 22–110). In this study, Cronbach’s α = 0.87 (total) and 0.71–0.87 (subscales) [17].

#### Data analysis

Data were entered and analyzed using IBM SPSS Statistics (v. 26). Prior to inferential testing, parametric assumptions were verified:

- Normality: Assessed via Shapiro–Wilk tests and Q–Q plots of residuals. All regression and GLM models met the normality assumption (*p* > 0.05).
- Homoscedasticity: Confirmed via visual residual scatterplots.
- Multicollinearity: Variance inflation factor (VIF) < 2.5 for all predictors.

Analytical steps included:

- Descriptive statistics (frequencies, %, *M*, *SD*, min/max) for demographics and key variables.
- Group comparisons: Independent *t*-tests (2 groups) and one-way ANOVA (≥ 3 groups).
- Bivariate associations: Pearson’s *r*.
- Prediction modeling: Hierarchical multiple linear regression, entering demographic variables (gender, academic term, age level) in Block 1, and AI use (AITOTAL) in Block 2. Effect size reported as *ΔR²*.
- Interaction effects: Univariate General Linear Model (GLM) with AITOTAL as covariate, and gender, age level, and term as fixed factors.
- Subscale analysis: Simple linear regressions per CPQ domain.

Statistical significance was set at *p* < 0.05 (two-tailed).

Missing data: Of 141 invited students, 7 questionnaires were excluded due to > 20% missing items. The final sample = 134 (response rate = 95.0%). For remaining items, ≤ 5% missing data occurred sporadically and were handled via pairwise deletion after confirming data were missing completely at random (MCAR) using Little’s MCAR test (χ² = 12.34, *df* = 15, *p* = 0.654).

Ethical Considerations.

The study was approved by the Research Ethics Committee of Abadan University of Medical Sciences (IR.ABADANUMS.REC.1403.134). Written informed consent was obtained from all participants. All procedures adhered to the Declaration of Helsinki. Participation was voluntary, confidential, and participants could withdraw at any stage without academic penalty.

## Results

This study included 134 undergraduate nursing students from Abadan University of Medical Sciences in 2024. The sample comprised 77 (57.5%) females and 57 (42.5%) males, with a relatively even distribution across academic terms 3 to 6. In terms of age, 35 students (26.1%) were under 19 years, 85 (63.4%) were aged 19–25 years, and 14 (10.4%) were over 25 years.

Descriptive Statistics.

Descriptive statistics for the main study variables are presented in Table 1. The mean total clinical performance score was 190.58 (*SD* = 11.95), ranging from 148.00 to 220.00. The mean score for artificial intelligence (AI) use was 83.33 (*SD* = 16.77), with scores ranging from 49.00 to 110.00. Subscale scores for clinical performance indicated high performance in management and personal management, with lower but stable scores in academic and research domains.

**Table 1:** Descriptive Statistics of Key Variables (*N* = 134)

Variable	*M*	*SD*	95% CI	Min	Max
Clinical Total Score	190.58	11.95	[188.53–192.63]	148.00	220.00
AI Use (AITOTAL)	83.33	16.77	[80.45–86.21]	49.00	110.00
Management Score	74.47	4.92	[73.62–75.32]	62.00	85.00
Academic Score	34.83	2.74	[34.36–35.30]	28.00	42.00
Personal Management Score	47.30	4.18	[46.58–48.02]	36.00	55.00
Patient-Centered Care	17.16	2.04	[16.81–17.51]	12.00	22.00
Research Score	16.82	2.08	[16.46–17.18]	11.00	22.00

*Note.* *CI* Confidence Interval (95%). All scores are based on validated clinical evaluation tools.*

### Correlation analysis

Pearson correlation analysis revealed a significant positive relationship between AI use and overall clinical performance, *r* = 0.424, *p* < 0.001. AI use was also significantly correlated with all clinical subscales (*p* < 0.05 for all), with the strongest associations observed for Research (*r* = 0.408, *p* < 0.001) and Patient-Centered Care (*r* = 0.383, *p* < 0.001). The weakest, though still significant, correlation was with Academic Score (*r* = 0.199, *p* = 0.021). These results suggest that higher engagement with AI is consistently linked to better performance across multiple domains of clinical competence.(Table 2).

**Table 2:** Pearson correlation matrix of study variables (*N* = 134)

Variable	1	2	3	4	5	6	7
Clinical Total	1						
AITOTAL	0.424	1					
Management	0.501	0.284	1				
Academic	0.316	0.199	0.245	1			
Personal Mngmt	0.555	0.356	0.418	0.278	1		
Pt-Cent Care	0.539	0.383	0.392	0.256	0.442	1	
Research	0.499	0.408	0.321	0.268	0.375	0.434	1

### Multiple linear regression

A hierarchical multiple linear regression was conducted to evaluate the incremental contribution of AI use to clinical performance, after controlling for demographic variables. In Block 1, age, gender, and academic term collectively explained only 2.9% of the variance in clinical performance (R² = 0.029, F(3, 130) = 1.310, *p* = 0.274), indicating no significant effect of demographics alone. The addition of AI use (AITOTAL) in Block 2 significantly increased the explained variance by ΔR² = 0.182 (F-change(1, 129) = 29.707, *p* < .001), yielding a final model accounting for 21.1% of the variance (R² = 0.211, adjusted R² = 0.187, F(4, 129) = 8.626, *p*< .001). In the final model, only AITOTAL was a significant predictor (β = 0.432, *p *< .001), confirming its robust and independent association with clinical performance* (*Table 3*).*

**Table 3:** Multiple linear regression predicting clinical performance (*N* = 134)

Predictor	*B*	*SE*	95% CI for *B*	β	*t*	*p*
Block1						
(Constant)	Term	7.95	[169.08–200.50]	----	23.24	< 0.001
Age Level	−0.13	0.35	[−0.82–0.56]	−0.03	−0.37	0.712
Gender	−0.41	1.96	[−4.29–3.47]	−0.02	−0.21	0.835
Term	−0.32	0.87	[−2.04–1.40]	−0.03	−0.37	0.712
Block 2						
(Constant)	184.37	9.95	164.72–204.02]	18.53	—	< 0.001
AITOTAL	0.308	0.057	[0.196–0.420]	0.432	5.450	< 0.001
Age Level	−0.627	0.336	[−1.29–0.036]	−0.147	−1.868	0.064
Gender	−1.818	1.907	[−5.58–1.95]	−0.076	−0.954	0.342
Term	−0.637	0.845	[−2.31–1.03]	−0.060	−0.754	0.452

*Note.* Hierarchical regression: Block 1 (demographics): *R²* = 0.029; Block 2 (+ AITOTAL): *R²* = 0.211, **ΔR² = 0.182**, *F*-change(1,129) = 29.707, *p* < 0.001

### Multivariate Analysis (UNIANOVA)

A univariate general linear model (GLM) was conducted with AI use (AITOTAL) as a covariate and age level, gender, and academic term as between-subjects factors. As shown in Table 3, there was a significant main effect for AITOTAL, *F*(1, 111) = 19.672, *p* < 0.001, indicating that higher AI use is associated with better clinical performance, independent of demographic variables.

No other main effects or interaction terms were statistically significant (all *p* > 0.05), reinforcing the unique contribution of AI use to clinical outcomes. (Table 4)

**Table 4:** Tests of Between-Subjects effects for clinical performance (*N* = 134)

Source	*SS*	*df*	*MS*	*F*	*p*
AITOTAL	2330.95	1	2330.95	19.67	< 0.001
Age Level	168.24	2	84.12	0.710	0.494
Gender	83.74	1	83.74	0.707	0.402
Term	178.75	3	59.58	0.503	0.681
Age Level × Gender	9.28	2	4.64	0.039	0.962
Age Level × Term	156.70	6	26.12	0.220	0.970
Gender × Term	450.84	3	150.28	1.268	0.289
Age Level × Ge × Ter	431.74	4	107.93	0.911	0.460
Error	13,152.	111	118.49	—	—
Corrected Total	18,990.	133	—	—	—

*Note.* Dependent variable: Clinical Total Score. *SS* = Sum of Squares; *MS* = Mean Square. *R²* = 0.307, Adjusted *R²* = 0.170

### Regression analysis on clinical subscales

To examine the specific impact of AI use on different domains of clinical performance, simple linear regressions were conducted for each subscale. As shown in Table 5, AI use significantly predicted performance in all five subscales (*p* < 0.05 for all).

**Table 5:** Simple Linear Regression Predicting Clinical Performance Subscales from AI Use (*N* = 134)

Clinical Subscale	*R²*	*B*	*SE*	*t*	*p*
Research	0.166	0.050	0.010	5.127	< .001
Patient-Centered Care	0.146	0.047	0.010	4.759	< .001
Personal Management	0.127	0.089	0.020	4.380	< .001
Management	0.081	0.084	0.024	3.422	.001
Academic	0.040	0.033	0.014	2.334	.021

*Note.* *B* = unstandardized regression coefficient; *SE* = standard error

The strongest effects were observed for Research (*R²* = 0.166, *p* < 0.001) and Patient-Centered Care (*R²* = 0.146, *p* < 0.001), followed by Personal Management (*R²* = 0.127, *p* < 0.001). The weakest, though still significant, effect was for Academic Score (*R²* = 0.040, *p* = 0.021).

These findings indicate that AI engagement is most strongly associated with competencies involving critical thinking, evidence-based practice, and interpersonal care. 

## Discussion

The findings of this study provide compelling evidence for the significant and positive association between artificial intelligence (AI) use and clinical performance among nursing students, independent of key demographic variables such as gender, academic term, and age. In a sample of 134 undergraduate nursing students from Abadan Nursing College, AI utilization emerged as the sole significant predictor of overall clinical performance in a multiple regression model, accounting for 21.1% of the variance. Notably, the strength of this relationship (β = 0.425, *p* < 0.001) underscores the transformative potential of AI integration in clinical education, aligning with the growing body of literature emphasizing technology-enhanced learning in healthcare training ([17]– [18]). Our findings align closely with the perceived usefulness (PU) construct of the Technology Acceptance Model (TAM). The significant association between AI use and clinical performance (β = 0.432, *p* < 0.001), particularly in research (R² = 0.166) and patient-centered care (R² = 0.146), suggests that students perceive AI as useful for enhancing evidence-based practice and interpersonal care skills. This is consistent with TAM’s proposition that users adopt technology when they believe it improves task performance.

Furthermore, the perceived ease of use (PEOU) dimension is reflected in the positive impact on personal management (R² = 0.127), indicating that AI tools help students organize clinical workflows, manage time effectively, and reduce cognitive load. The non-significant effects of demographic variables further support TAM’s assertion that technology acceptance is primarily driven by functional utility rather than individual characteristics.

The observed correlation between AI use and clinical competence (*r* = 0.424) is both statistically and educationally meaningful. This result resonates with recent studies indicating that AI-powered tools—such as virtual patient simulators, intelligent tutoring systems, and predictive analytics in clinical decision-making—enhance students’ critical thinking, diagnostic reasoning, and procedural confidence [19–22]. For instance, AI-driven simulation platforms have been shown to personalize learning trajectories, offering real-time feedback and adaptive scenarios that mirror complex clinical environments [23]. It is plausible that students with higher AI engagement in this study benefited from such tools, leading to improved performance across structured clinical assessments. While our study demonstrates a robust positive association between AI use and clinical performance, it is important to acknowledge that not all prior research has reported significant effects. For instance, Al-Zahrani and Alasmari (2025) found no significant correlation between AI readiness and academic performance among nursing students in Saudi Arabia (*r* = 0.12, *p* = 0.18) [8]. Similarly, a systematic review by Dehghani et al. (2025) reported mixed findings across Middle Eastern studies, with 40% showing non-significant AI-performance relationships [14].

These discrepancies may be attributed to contextual and methodological differences: [1] Studies measuring AI readiness or acceptance (rather than actual use) may not capture behavioral outcomes; [2] In settings with limited AI infrastructure (as in 58% of Middle Eastern nursing programs per Mohamed et al., 2025), perceived usefulness may not translate to actual usage; [3] Cultural factors, such as faculty resistance or traditional pedagogical approaches, may moderate AI’s impact. Our study’s focus on actual AI use rather than intention, combined with Iran’s unique educational context, likely explains the stronger associations observed here [11].

Importantly, the multivariate analysis confirmed the robustness of AI’s impact, revealing a significant main effect even after controlling for potential confounders. The absence of significant effects for gender, academic term, and age level suggests that the benefits of AI are broadly accessible across diverse learner profiles. This egalitarian potential is particularly promising in resource-constrained educational settings, where AI may help mitigate disparities in access to high-quality clinical training ([24]– [25]). The non-significant interaction effects further indicate that AI’s influence is consistent across different demographic subgroups, reinforcing its role as a universal enhancer of clinical learning.

A deeper exploration of subscale performance revealed that AI use had the strongest predictive power for Research (*R²* = 0.166), Patient-Centered Care (*R²* = 0.146), and Personal Management (*R²* = 0.127). These findings are particularly insightful. The strong link with research performance suggests that students who actively engage with AI are more likely to utilize data-driven approaches, access evidence-based resources efficiently, and develop analytical skills critical for evidence-based practice ([26]– [27]). This aligns with studies showing that AI-powered literature review tools and data analysis assistants significantly reduce the cognitive load on students, enabling deeper engagement with research content ([28]– [29]).

The significant association with patient-centered care is equally noteworthy. AI tools that simulate patient interactions, analyze communication patterns, or provide emotional intelligence feedback may foster empathy, active listening, and individualized care planning—core components of patient-centered nursing [30]. Similarly, the impact on personal management—which includes time management, self-regulation, and organizational skills—suggests that AI-based scheduling, task prioritization apps, and reflective learning platforms may support students in managing the cognitive and emotional demands of clinical training ([31]– [32]).

Even the weakest, yet still significant, effect on the Academic subscale (*R²* = 0.040) implies that AI contributes to foundational knowledge acquisition, possibly through intelligent flashcards, automated quiz generation, or natural language processing-based tutoring systems [33]. While the effect size is modest, it reflects the complementary role of AI in reinforcing didactic learning alongside experiential clinical training.

These findings collectively advocate for the systematic integration of AI literacy into nursing curricula. As the World Health Organization ([34]– [35]) emphasizes in its *Global Strategy on Digital Health*, preparing future healthcare professionals to leverage AI ethically and effectively is no longer optional but essential. Curricular reforms should include training in AI fundamentals, data interpretation, algorithmic bias awareness, and the ethical use of AI in patient care ([36]– [37]). Furthermore, institutions must invest in equitable access to AI tools to ensure all students benefit, regardless of socioeconomic or demographic background.

### Strengths and limitations

A key strength of this study is its use of multivariate modeling to isolate the effect of AI use from demographic confounders, enhancing the validity of the observed relationships. The inclusion of multiple clinical performance domains also provides a nuanced understanding of AI’s impact. However, several limitations should be acknowledged. First, the cross-sectional design precludes causal inference; longitudinal or experimental studies are needed to establish directionality. Second, AI use was measured via self-report, which may be subject to recall or social desirability bias. Future studies should incorporate objective metrics such as AI platform usage logs or digital footprints. Third, the sample was drawn from a single institution in Iran, limiting generalizability. Multi-center, cross-cultural studies are warranted to validate these findings in diverse educational contexts.

### Implications for practice and research

Educators and policymakers should consider embedding AI-enhanced learning modules into clinical rotations and simulation labs. Pilot programs integrating AI tutors or virtual patients could be evaluated for their impact on student outcomes. From a research perspective, Future research should adopt mixed-methods designs to triangulate our quantitative findings. Specifically:

(1) Experimental interventions testing specific AI modules (e.g., virtual patient simulators for diagnostic reasoning, AI chatbots for reflective practice) in randomized controlled trials;

(2) Longitudinal studies tracking how AI engagement during education influences professional practice and patient outcomes post-graduation;

(3) Qualitative exploration of barriers to AI adoption in resource-constrained settings, including faculty perspectives and curriculum integration challenges;

(4) Cross-cultural comparative studies examining how cultural values (e.g., collectivism vs. individualism) and educational policies moderate AI’s impact on clinical learning.

As recommended by the WHO Global Strategy on Digital Health (2024), such research should prioritize equitable access and ethical implementation, ensuring AI tools do not exacerbate existing disparities in nursing education quality [36].

## Data Availability

The anonymized dataset and analysis code are available upon reasonable request.
